# Interments in the City and Liberties of Philadelphia, from the 11th to the 18th of December

**Published:** 1841-12-25

**Authors:** 


### HEALTH OF THE CITY.

Interments *in the City and Liberties of Philadelphia, from the 11th to the 18th of December.*

| Diseases. | Adults. | Children. | Diseases. | Adults. | Children. |
|---|---|---|---|---|---|
| Apoplexy | 1 | 0 | *Brought forward,* | 30 | 34 |
| Burns, | 1 | 0 | Mania a Potu, | 1 | 0 |
| Consumption of | | | Old age, | 1 | 0 |
| the lungs, | 7 | 4 | Palsy, | 2 | 0 |
| Convulsions, | 0 | 2 | Pneumothorax, | 4 | 0 |
| Dropsy, | 2 | 0 | Scrofula, | 0 | 1 |
| —— Head, | 0 | 3 | Small pox, | 3 | 5 |
| Disease of the | | | Stone, | 1 | 0 |
| heart, | 1 | 0 | Still-born, | 0 | 4 |
| Debility | 0 | 2 | Unknown, | 2 | 1 |
| Epilepsy, | 2 | 0 | Varioloid, | 1 | 0 |
| Erysipelas, | 0 | 3 | Violence, | 1 | 0 |
| Effusion on chest, | 1 | 0 | | — | — |
| Fever, | 0 | 2 | Total, 91— | 46 | 45 |
| —— Congestive, | 0 | 1 | | | |
| —— Typhus, | 2 | 0 | Of the above, there | | |
| —— Puerperal, | 1 | 0 | were under 1 year, | | 23 |
| —— Scarlet, | 0 | 4 | From 1 to 2 | | 8 |
| Hernia, | 1 | 0 | 2 to 5 | | 8 |
| Inflammation of | | | 5 to 10 | | 5 |
| the Brain, | 3 | 1 | 10 to 15 | | 0 |
| —— Bronchi, | 0 | 4 | 15 to 20 | | 1 |
| —— Lungs, | 5 | 1 | 20 to 30 | | 12 |
| —— Stomach, | 1 | 0 | 30 to 40 | | 2 |
| —— Bowels, | 0 | 2 | 40 to 50 | | 9 |
| —— Breast, | 0 | 2 | 50 to 60 | | 5 |
| —— Peritonæum, | 1 | 0 | 60 to 70 | | 4 |
| Marasmus, | 0 | 1 | 70 to 80 | | 7 |
| Malformation, | 0 | 1 | 80 to 90 | | 5 |
| Measles, | 0 | 1 | 90 to 100 | | 2 |
| Mortification, | 1 | 0 | | | —— |
| | — | — | Total, | | 91 |
| *Carried forward,* | 30 | 34 | | | |

Of the above there were 5 from the almshouse, and 7 people of colour, which are included in the total amount.

